# Structural characterization of a mixed-linkage glucan deficient mutant reveals alteration in cellulose microfibril orientation in rice coleoptile mesophyll cell walls

**DOI:** 10.3389/fpls.2015.00628

**Published:** 2015-08-18

**Authors:** Andreia M. Smith-Moritz, Zhao Hao, Susana G. Fernández-Niño, Jonatan U. Fangel, Yves Verhertbruggen, Hoi-Ying N. Holman, William G. T. Willats, Pamela C. Ronald, Henrik V. Scheller, Joshua L. Heazlewood, Miguel E. Vega-Sánchez

**Affiliations:** ^1^Physical Biosciences Division, Lawrence Berkeley National Laboratory, Joint BioEnergy Institute, Berkeley, CAUSA; ^2^Lawrence Berkeley National Laboratory, Berkeley Synchrotron Infrared Structural Biology Program, Berkeley, CAUSA; ^3^Department of Plant and Environmental Sciences, University of Copenhagen, CopenhagenDenmark; ^4^Department of Plant Pathology, UC Davis Genome Center, University of California, Davis, Davis, CAUSA

**Keywords:** type II cell walls, cellulose, FT-MIR spectroscopy, mixed-linkage glucan, primary cell wall, rice

## Abstract

The *CELLULOSE SYNTHASE-LIKE F6* (*CslF6*) gene was previously shown to mediate the biosynthesis of mixed-linkage glucan (MLG), a cell wall polysaccharide that is hypothesized to be tightly associated with cellulose and also have a role in cell expansion in the primary cell wall of young seedlings in grass species. We have recently shown that loss-of-function *cslf6* rice mutants do not accumulate MLG in most vegetative tissues. Despite the absence of a structurally important polymer, MLG, these mutants are unexpectedly viable and only show a moderate growth compromise compared to wild type. Therefore these mutants are ideal biological systems to test the current grass cell wall model. In order to gain a better understanding of the role of MLG in the primary wall, we performed in-depth compositional and structural analyses of the cell walls of 3 day-old rice seedlings using various biochemical and novel microspectroscopic approaches. We found that cellulose content as well as matrix polysaccharide composition was not significantly altered in the MLG deficient mutant. However, we observed a significant change in cellulose microfibril bundle organization in mesophyll cell walls of the *cslf6* mutant. Using synchrotron source Fourier Transform Mid-Infrared (FTM-IR) Spectromicroscopy for high-resolution imaging, we determined that the bonds associated with cellulose and arabinoxylan, another major component of the primary cell walls of grasses, were in a lower energy configuration compared to wild type, suggesting a slightly weaker primary wall in MLG deficient mesophyll cells. Taken together, these results suggest that MLG may influence cellulose deposition in mesophyll cell walls without significantly affecting anisotropic growth thus challenging MLG importance in cell wall expansion.

## Introduction

Plant cells are surrounded by walls, which are unique and dynamic structures necessary for normal growth and development. They are also involved in mechanical support and play roles in protection against biotic and abiotic stresses. A typical primary plant cell wall is mainly composed of cellulose and matrix polysaccharides (hemicelluloses and pectin; Carpita and Gibeaut, 1993). In vascular plants, three types of primary cell walls have been described. Type I primary cell walls are comprised of a cellulose-xyloglucan framework surrounded by pectic polysaccharides and other hemicelluloses, and are found in most flowering plants (Carpita and Gibeaut, 1993). Type II primary walls, which are found in commelinid monocots are composed of cellulose microfibrils embedded in a glucuronoarabinoxylan network with little pectin and xyloglucan content (Carpita, 1996; Fincher, 2009). Type II walls in Poales (grasses and some closely related species) also contain mixed-linkage glucan (MLG) as a major component in addition to glucuronarabinoxylan and cellulose. Recently, Silva et al. (2011) have presented a Type III primary cell wall associated with ferns and characterized by a predominance of mannan and a low pectic content. The compositional differences between these cell wall types have been well documented (Carpita and Gibeaut, 1993; Carpita, 1996). However, the organization of cell wall polysaccharides and the variation in their structure are less understood. As a result, multiple cell wall models have been proposed that differ by the number and types of interactions that exist among all the components of cell walls (Cosgrove, 2000).

Recent interest in bioenergy and the need to obtain fermentable sugars from biomass has highlighted the importance of understanding the overall structure, organization, and dynamic processes that govern the formation of the plant cell wall. This has been especially relevant for target biofuel crops such as the grass species *Miscanthus* and switchgrass, which have type II cell walls. Potential biomass crops must have cell walls that are structurally sound to withstand the turgor pressure forces in varying environmental conditions, and that are flexible enough to allow relatively easy reorganization during growth and cell elongation phases. It is generally agreed that cellulose microfibrils are the primary load bearing components in the plant cell wall (Buckeridge et al., 2004). However, there is no consensus on how other abundant polysaccharides such as xyloglucan, arabinoxylan, or MLG are organized or linked with the cellulose microfibrils. For Type I cell walls, the most widely referenced model is the tethered network model first proposed by Fry (1989) and by Hayashi (1989), and which has been subsequently updated by various groups. In this model, cellulose microfibrils are coated and crosslinked with xyloglucan and surrounded with pectin and other hemicelluloses (Somerville et al., 2004). However, this model has been revised based on the observation that the xyloglucan-deficient mutants are viable and only show minor growth reductions, albeit with weakened primary walls. These findings suggest an alternative role for xyloglucan than as a tether for cellulose microfibrils (Cavalier et al., 2008; Park and Cosgrove, 2012).

A model for type II cell walls of grasses was also developed based on various polysaccharide sequential extraction experiments, spectromicroscopy analysis, as well as high resolution imaging techniques such as electron microscopy of the maize coleoptile epidermal primary wall (Carpita et al., 2001). In this model, cellulose microfibrils are coated by MLG, arabinoxylan with low arabinosyl substitutions and glucomannan, which are embedded in a matrix of pectins, highly substituted arabinoxylan and glucomannan. In a recent study, Kiemle et al. (2014) showed that MLG binds to both cellulose and arabinoxylan *in vitro* and that based on a biomechanical creep test, MLG does not seem to act as a wall tether. Additionally, Kozlova et al. (2014) proposed that MLG serves as a gel-like filler between cellulose and glucuronoarabinoxylan in elongating root tissue. Taken together, these results challenge the cell wall models involving the tethering of cellulose microfibrils by hemicellulosic polysaccharides in both Type I and Type II primary walls.

Mixed-linkage glucan, a linear polymer composed of glucose monomers linked by β-1,3 and β-1,4 glycosidic linkages, is in itself an interesting cell wall polysaccharide since it is not present in Type I walls and, at least in maize and barley coleoptiles, accumulates during phases of rapid elongation and is then hydrolyzed after peak growth (Carpita, 1984; Gibeaut et al., 2005). Interestingly, MLG is also found abundantly in tissues no longer expanding and in lignified, secondary cell walls in rice and other grasses (Vega-Sánchez et al., 2012), indicating that the polymer is not exclusively associated with growth. MLG is also highly abundant in endosperm cell walls in the grains of certain cereals, such as barley and *Brachypodium* (Wilson et al., 2006; Guillon et al., 2011). The recent availability of rice MLG-deficient mutants (Vega-Sánchez et al., 2012) has made it possible to test the function(s) of this polysaccharide in the grass cell wall. MLG-deficient mutants *cslf6-1* and *cslf6-2* are loss-of-function mutants in the rice *CslF6* gene, which is required for MLG accumulation in vegetative tissues (Vega-Sánchez et al., 2012). These mutants have a drastic decrease in MLG content (97% reduction in developing leaves and virtually undetectable in other tissues) and yet do not display altered morphological phenotypes typically associated with mutations affecting primary cell wall development, other than moderately stunted growth (Vega-Sánchez et al., 2012). These mutants are ideal candidates to test MLG proposed role, for the first time *in vivo*.

To better understand the role of MLG in grasses, we have sought to characterize the composition and structural changes in the cell wall associated with the lack of MLG in rice. Using a combination of biochemical and biophysical methods, including high-resolution spectromicroscopy via synchrotron radiation, we were able to analyze both bulk and cell-specific changes in the primary cell walls of 3 day-old etiolated rice wild type and mutant seedlings. Our results show that, although little changes in cell wall composition occur in response to MLG deficiency, the deposition of cellulose microfibrils is affected in *cslf6* mutant mesophyll cells with no measurable difference in bending rigidity of fresh tissue. Our data highlight the plastic nature of the plant cell wall and reveal that Type II walls in 3 day-old seedlings do not require MLG for growth and cell elongation.

## Materials and Methods

### Plant Material and Growth Conditions

Rice (*Oryza sativa* L.) *cslf6* mutant and corresponding wild type (cultivar Nipponbare) were used in this study. De-husked seeds were first sterilized in a 50% v/v Clorox solution for 20 min, followed by 10 washes with autoclaved deionized water. Seeds were then placed in tissue culture plastic cups containing growth medium (1/2 strength Murashige and Skoog basal salt mixture, 30 g/L sucrose, and 1.5 g/L Phytagel) for germination and growth in the dark at 28°C for 3 days.

### Preparation of Plant Material for Microscopy, FT-MIR Spectroscopy, and Imaging

Three day old seedlings were fixed for 24 h at 4°C in 4% w/v formaldehyde in 50 mM piperazine-N,N′-bis(2-ethanesulphonic acid), 5 mM EGTA, 5 mM MgSO_4_, pH 6.9 as described in Verhertbruggen et al. (2009). Seedlings were embedded in 7% w/v agarose and sectioned (60 μm for microscopy, 30 μm for FT-MIR spectroscopy) using a Leica VT1000S vibratome. At least 5–10 biological replicates were used per genotype.

### Comprehensive Microarray Polymer Profiling (CoMPP) Analysis

Comprehensive Microarray Polymer Profiling (CoMPP) was carried out as described in a previous publication (Moller et al., 2008). Briefly rice cell wall material was sequentially extracted with 50 mM diamino-cyclo-hexane-tetra-acetic acid (CDTA) to first solubilize pectin, followed by a strong base, 4 M NaOH with 0,1% v/v NaBH_4_, to extract hemicelluloses. Three-hundred microliters for 10 mg cell wall material were used for each extraction and shaken on a Retsch TissueLyser at 6/s for 2 h for both CDTA and NaOH. The extracts for wild-type and mutant cell walls were subsequently spotted on nitrocellulose membranes with a pore size of 0.45 μm (Whatman, Maidstone, UK) using an Arrayjet Sprint (Arrayjey, Roslin, UK). Each sample was printed with three dilutions and four technical replicates. The arrays were probed with a library of plant cell wall specific monoclonal antibodies for determining the relative sugar abundances. Arrays were incubated for 2 h with the primary antibody, washed three times with phosphate-buffered saline (PBS; 140 mM NaCl, 2.7 mM KCl, 10 mM Na_2_HPO_4_, 1.7 mM KH_2_PO_4_, pH. 7.5) and probed with secondary antibodies conjugated to alkaline phosphatase for visualization for 2 h as well. The arrays were developed a BCIP/NBT (5-bromo-4-chloro-3′-indolyphosphate/nitro-blue tetrazolium chloride) substrate. The developed arrays were scanned in a Canon 9950F scanner^1^ and each spot was converted to a value based on pixel intensity using Array-Pro Analyzer 6.3 (Media Cybernetics, Rockville, MD, USA). The average of the 12 values representing a sample was calculated and forms the basis of one number in the heatmap. Signal counts across the entire sample set were then normalized to the highest value in the dataset and a cut-off of 5 was introduced. For our experiment, (1–3; 1–4)-β-D-glucan antibody labeling (MLG) for NaOH extracted wild type (NPB) sample had the highest fluorescent count and the array was normalized accordingly.

### Monosaccharide Composition Analysis

Cell wall monosaccharide composition following trifluoroacetic acid (TFA) hydrolysis (2 M TFA at 121°C for 1 h) was determined from de-starched alcohol insoluble residues (AIR) of 3-day-old etiolated seedlings using high performance anion-exchange chromatography according to a method previously described (Vega-Sánchez et al., 2012).

### Derivatization of Cell Wall Components for Linkage Analysis

Cell wall preparations were methylated by the NaOH method of Ciucanu and Kerek (1984) and according by Burton et al. (2000) and Cocuron et al. (2007). Approximate 1 mg of AIR was transferred to a glass tube with a lid and a small magnetic bar. Then, 200 μL of dry DMSO was added using a dry glass pipette, and the solution was sonicated for 5 min and stirred for 16 h. Two-hundred microliters of freshly prepared NaOH/DMSO (an initial 50% (v/v) solution was centrifuged to remove the water residue and to ensure a water-free alkali solution) was added to approximately 100 mg mL^-1^ and stirred for another 15 min. Subsequently, methylation was carried out by adding 150 μL of iodomethane in a nitrogen atmosphere. The solution was briefly vortexed, stirred for 1 h and the process was repeated once. After an additional 3 h, the reaction mixture was quenched with 2 mL of water and bubbled with nitrogen until the solution became clear. Finally, the reaction was extracted with 2 mL of dichloromethane (CH_2_Cl_2_) and centrifuged at 180 ×*g* for 2 min. The aqueous phase was removed and the reaction was washed with 3 mL water, until the samples reached a pH value of (6.0–6.5). After evaporation of CH_2_Cl_2_ residue under a nitrogen stream at 40°C, 10 μg of inositol was added to the partially methylated polysaccharides as an internal standard and the samples were hydrolyzed in 250 μL of 2 M TFA at 121°C for 90 min. The TFA was evaporated under a stream of air at 40°C, washed twice with 300 μL of 2-propanol and evaporated each time. The monosaccharides were reduced at the corresponding anomeric carbon by adding 200 μL of 10 mg mL^-1^ sodium borodeuteride (NaBD_4_; Sigma) in 1 M ammonium hydroxide (NH_4_OH). After 1 h, the reductant was neutralized with 150 μL of glacial acetic acid. A volume of 250 μL acetic acid:methanol anhydride (1:9 v/v) solution was added and evaporated until dry. This step was repeated three times. Subsequently, the precipitate was washed four times with 250 μL of methanol and dried by evaporation. Acetylation of the partially methylated alditols was carried out by adding 50 μL of acetic anhydride and 50 μL of pyridine. The reaction was incubated for 20 min at 121°C, cooled down to room temperature and evaporated under a light stream of air. The samples were washed twice with 200 μL of toluene and evaporated under an air stream. The partially methylated alditol acetate derivatives were extracted with 1.2 mL of ethyl acetate and 5 mL of water, and centrifuged at 180 ×*g* for 2 min. The organic phase was transferred to a microcentrifuge tube and evaporated under a soft stream of air. The derivatized sugars were suspended in 100 μL of acetone and transferred into a gas chromatography glass vial.

### Linkage Analysis of Cell Walls

The derivatized sugars were separated using a Thermo Trace GC Ultra gas chromatography system (GC) with a capillary fused silica SP-2330 (30 m × 0.25 mm layer thickness of 20 microns internal diameter, Supelco^®^- Sigma–Aldrich) GC column and detected by mass spectrometry (MS). After an initial temperature of 80°C for 3 min, the temperature was gradually increased to 160°C at a rate of 30°C/min, then increased gradually to 210°C at a rate of 2°C/min, and finally increased to 240°C at a rate of 5°C/min and held for 10 min. The separated permethylated alditol acetates (PMAA) were detected by a Finnigan Polaris Q mass spectrometer (Thermo) and analyzed using the XcaliburTM Data System. Identification of the derivatives and deduction of the glycosidic linkages were based on the mass spectrum and retention time of previously established standards, and by its ion patterns according to the spectral database PMAA of the Complex Carbohydrate Research Center at the University of Georgia, USA (CCRC) and as described by (Carpita and Shea, 1989) for interpreting data derived from the analysis of linkages. The described PMAA analyses were performed in triplicates and the results were averaged. The results are presented as the percentage of composition of each sample, calculated by normalizing to the total ion chromatogram peak areas identified.

### FTIR Time Course Study of Arabinoxylan Substitutions

To study the development of arabinoxylan substitutions over the time, samples were collected every 8 h starting at 48 h post germination until the leaf emerged from the coleoptiles (68 h post-germination for both wild type and mutant). Samples were immediately fixed, as stated above in preparation of plant material section, and embedded in agarose for sectioning. Rice coleoptiles were sectioned with a vibratome to a thickness of 30 μm, placed on barium fluoride windows and dried. Infrared spectra were taken in transmission mode with a Bruker Instruments Hyperion with a MCT detector in transmission mode. A 50 μm × 50 μm area was measured using a 36X microscope objective. At least 5–10 biological samples were measured at various mesophyll cell locations. The infrared spectra was smoothed then cropped from 800–1200 cm^-1^ before a second derivative was calculated. The data from all the mutant and wild type samples at all stages were processed using principal component analysis (PCA). The first principal component loading plot corresponded to peaks previously assigned to arabinoxylan substitutions (Robert et al., 2005; Saulnier et al., 2009). Plotting the first principal component score versus the second principal component score allows arabinoxylan substitution comparison.

### Crystalline Cellulose Determination

Crystalline cellulose content was determined from TFA insoluble AIR fractions using the Updegraff method (Updegraff, 1969). Briefly, the insoluble pellet was washed with H_2_O and acetone and dried overnight in a vacuum concentrator. The dried pellet was re-suspended in 67% v/v H_2_SO_4_ shaking at room temperature for 1 h. The sample was clarified at 20,000 ×*g* and diluted with 0.2% w/v anthrone (Sigma–Aldrich) in concentrated H_2_SO_4_ and incubated in boiling water for 5 min. Glucose concentration was measured using a spectrophotometer at λ = 620 nm against a standard curve prepared with Avicel (Sigma–Aldrich).

### Fluorescence Microscopy

Sections were successively incubated at room temperature in a blocking solution [5% w/v milk powder protein/phosphate-buffered saline (PBS) and 0.05% v/v Tween solution] for 1 h and in solutions containing primary probes for 1 h. The dilutions of probes in 5% w/v milk/PBS/Tween solution were as follows: 1:200X dilution for Anti-α-Tubulin (Invitrogen), and 1:1000X dilution for the cellulose binding module CBM3a. Sections for anti-α-tubulin labeling were extensively washed (5 min, three times) then incubated in milk powder protein/PBS solution containing the FITC conjugated secondary antibody for 1 h followed by another extensive wash. Sections for CBM3a detection were incubated in 1:1000X anti-histidine antibody in buffer solution for 1 h. After an extensive wash, the sections were incubated in the presence of FITC conjugated secondary antibody for 1 h and washed again. Sections incubated without primary antibodies were used as negative controls. Wild type sections treated with lichenase (from *Bacillus* sp., Megazyme; 1:1000 dilutions) for 1 h prior to CBM3a labeling were also used as controls. Brightness and contrast adjustments were done using ImageJ (NIH, Bethesday, MD, USA^2^, 1997–2014).

### Polarized Fourier Transformed Infrared Spectroscopy

A Hyperion FT-MIR Spectrometer (Bruker Optics) was used to obtain spectra from sectioned plant samples. Spectra were taken in transmission mode with a MIR polarizer. Spectral absorbance for sample replicates (3–5) of wild type and mutant, covering a range from 600 to 4000 cm^-1^, was taken at a spectral resolution of 4 cm^-1^. A total of 32 scans were taken for each sample and co-added to give the final spectra. Preprocessing of the absorption spectra was done using Opus software (Bruker Optics). Absorption spectra were cropped from 800 to 2000 cm^-1^, smoothed using a Savitzky–Golay filter with 13 points, and baseline corrected. The absorption values at 1156 cm^-1^ were recorded at polarizations perpendicular and parallel to cell orientation (direction of cell elongation). The dichroic ratio was calculated based on the absorption at 1156 cm^-1^ (parallel polarization value divided by the perpendicular value). All data processing was performed using custom scripts run on Matlab (Mathworks).

### High-Resolution FTIR Spectromicroscopy

All measurements were performed with a Nicolet FTIR bench at the infrared beamline of the Advanced Light Source (Lawrence Berkeley National Laboratory, Berkeley, CA, USA^3^). Three biological replicates of both wild type and mutant were measured and processed accordingly. Images and spectra presented are representative of the measurements we obtained. Spectral absorbance for sample replicates of wild type and mutant, covering a range from 600 to 4000 cm^-1^, was taken at a spectral resolution of 4 cm^-1^. A total of 128 scans were performed for each sample and co-added to give the final spectra. The first derivatives of the spectra were calculated using a Savitzky–Golay filter. This was done to deal with the changing baseline condition that occurs during the 8–9 h data acquisition window (Lasch, 2012; Trevisan et al., 2012). PCA was used for data compression and the first PC scores were plotted according to spatial coordinates, generating an image resembling the mesophyll cells. Areas of interest were identified by first principal component scores. The PC scores that represented 95% of the variation (between 8 and 9 PC scores) of the areas of interest were clustered using Gaussian Means (G-means; Hamerly and Elkan, 2003b), a variation of K-means. G-means performs K-means clustering but also incorporates a non-parametric test, Kolmogorov–Smirnov, to determine if clusters are separate discrete groups using a defined threshold level. This was done by the following sequence of steps. The data, comprising the first derivative of cropped spectra from 800–2000 cm^-1^, was first clustered into two groups using K-means with squared Euclidean distance. Then, the distance vector between the group means was calculated. Each member of the two groups is projected against this distance vector (the dot product) to determine the location in the distance vector plane. Using these new coordinates, the two groups were run through the Kolmogorov–Smirnov test to determine if they represent two separate populations. If the criteria is met, each of the groups was clustered with K-means into two smaller groups and tested again for uniqueness using the same threshold (Hamerly and Elkan, 2003a). Using this approach, we were able to define a criterion for the number of groups we can use when performing K-means clustering of the data sets. Once the numbers of groups were defined and members identified, PCA was performed on the first derivatives of the remaining areas. PC1 vs. PC2 were plotted and grouped according to their cluster ID to identify which principal component separates the groups. Following this method, the first PC loading was used to describe the difference between groups and therefore represents the chemical and structural differences reflected in the clustering according to spatial coordinates. All data processing was performed using custom scripts run on Matlab (Mathworks).

### Mechanical Strength Testing

Both mutant and wild-type rice seedlings were harvested and fresh samples were immediately tested. Prior to mechanical testing, the diameter of fresh samples and thawed frozen samples was measured using a dissection microscope. Three point bending testing using an Instron 5942 universal testing machine was carried out by centering the bottom 5 mm of the samples on a mini flex fixture with 3 mm span gap and with a 5N force transducer set at a crosshead speed of 1 mm/min until a distance of 2 mm was traveled. Subsequent force and distance were recorded and a stress/strain plot was generated. The linear portion of the curve was fitted and the slope calculated to determine the bending stiffness for fresh samples. The bending rigidity (R) was calculated as *R* = *L*^3^ (df/dy)/48, with L, span length, df/dy, slope of initial deformation.

### Coexpression Analysis

The analysis was performed *in silico* using the co-expression analysis tools from the Rice Oligonucleotide Array Database (ROAD), a public online resource^4^. The database and associated tools have been described previously (Cao et al., 2012). The co-expression tool uses Pearson correlation coefficient (PCC) cutoffs to identify co-expressed genes (Cao et al., 2012). Using the rice *CslF6* coding sequence as query and a PCC of 0.75, a list of *CslF6* co-expressed genes was generated. Among those genes, the loci for four primary cell wall *Cellulose Synthase* (*CesA*) genes were identified (*CesA1*, *CesA3*, *CesA6*, and *CesA8*). The normalized expression values (Log2 values) for each of these genes throughout 27 rice anatomical stages was plotted using the meta analysis tool available in the ROAD database, as described in Vega-Sánchez et al. (2012).

## Results

Unless otherwise noted, we used 3-day-old etiolated rice seedlings grown under sterile conditions in tissue culture media for all experiments. To make physiologically relevant comparisons between wild type and mutant seedlings, samples were collected at the growth stage where the developing leaf emerged from the coleoptile, which is associated with the cessation of coleoptile cell elongation (Frohlich et al., 1994). These seedlings represented a convenient model to study the effects of MLG deficiency due to the rapid rate of growth that occurs over a short period of time and the availability of tissues rich in primary cell walls.

### Matrix Polysaccharide Cell Wall Composition Analysis

**FIGURE 1:**
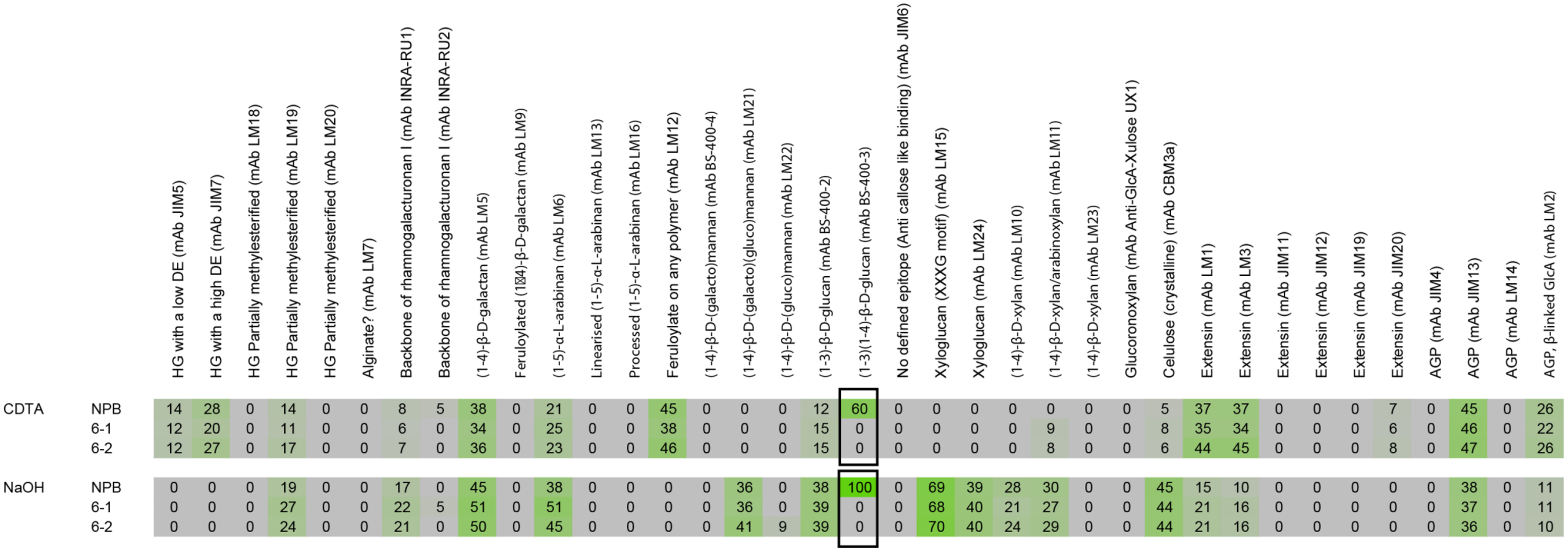
**Comprehensive microarray polymer profiling (ComPP) of wild type (NPB) and mutant lines *cslf6-1* and *cslf6-2***. Rice cell wall material was sequentially extracted with CDTA followed by NaOH. The extracts were then subsequently printed on nitrocellulose membranes and probed with a library of cell wall specific monoclonal antibodies to determine relative abundance. In our experiment, the (1–3), (1–4)-β-D-glucan antibody labeling (MLG) from NaOH extracted wild type (NPB) samples had the highest fluorescent count and the array was normalized accordingly in reference to the MLG signal. ComPP analysis showed significant difference in only MLG (boxed).

**FIGURE 2:**
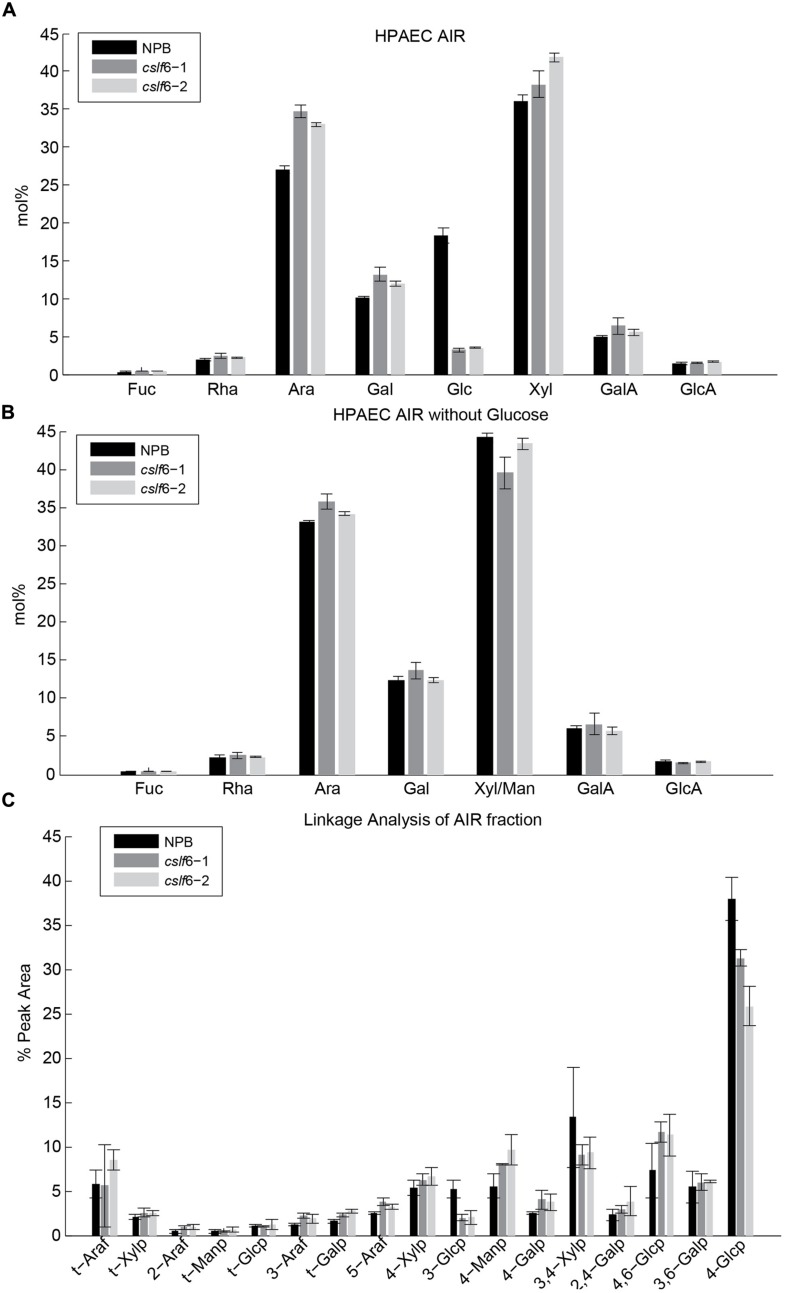
**Matrix polysaccharide sugar content analysis by High-Performance Anion-Exchange Chromatography (HPAEC) following Trifluoroacetic acid (TFA) hydrolysis.** HPAEC analysis on total alcohol insoluble residue (AIR) with **(A)** and without glucose **(B)**. **(C)** Cell wall linkage analysis of AIR. Error bars mark SD calculated from 3 biological replicates.

We previously analyzed the monosaccharide composition of non-cellulosic polysaccharides in AIR of 7 day-old seedlings and mature stems and found that sugars generally associated with pectin (galacturonic acid, rhamnose, and galactose) were increased in the *cslf6* mutants, especially in stems (Vega-Sánchez et al., 2012). In order to more accurately identify potential changes in cell wall composition in response to MLG deficiency in the primary wall of 3-day-old seedlings, we carried out a detailed analysis of the matrix polysaccharide fraction in total and sequentially extracted AIR samples of wild type (NPB) and *cslf6* mutants. Glycan microarray profiling [also known as CoMPP (Willats et al., 2002)] using a battery of antibodies that specifically recognize plant cell wall polysaccharides and glycoproteins, revealed little changes in the relative abundance of cell wall components. However, as expected, signals from the anti-MLG antibody were absent in the mutants (**Figure 1**). There were no other significant differences noted in either the pectin-rich (CDTA) fraction, or in the hemicellulosic-rich, NaOH fraction. To further confirm the CoMPP results, we performed both cell wall monosaccharide composition and linkage analyses of AIR. As expected, the *cslf6* mutants showed a large and significant decrease in TFA susceptible glucose (Glc) content (**Figure 2A**). However, the dramatic difference in glucose content could skew the results of the HPAEC analysis, so we also present the data as mol% of total monosaccharides without taking glucose into consideration (**Figure 2B**). When glucose was removed from the matrix monosaccharide analysis, no significant differences were observed between wild type and mutant (**Figure 2B**). We also performed monosaccharide analysis of sequentially extracted AIR samples with CDTA, sodium carbonate and 4 M NaOH to enrich for soluble pectin, covalently linked pectic polysaccharides and hemicelluloses, respectively. Both pectic and hemicellulosic fractions released glucose associated with MLG, as all fractions had significantly decreased glucose content in the mutants (Supplementary Figure S1). Linkage analysis showed only a decrease in both 3-linked and 4-linked glucose moieties in the analysis of neutral sugars most likely due MLG (**Figure 2C**). Taken together, the results from the matrix polysaccharide and CoMPP analyses showed that MLG deficiency, at least in these mutants, does not result in significant pleotropic changes in other cell wall components.

### Arabinoxylan Structure

**FIGURE 3:**
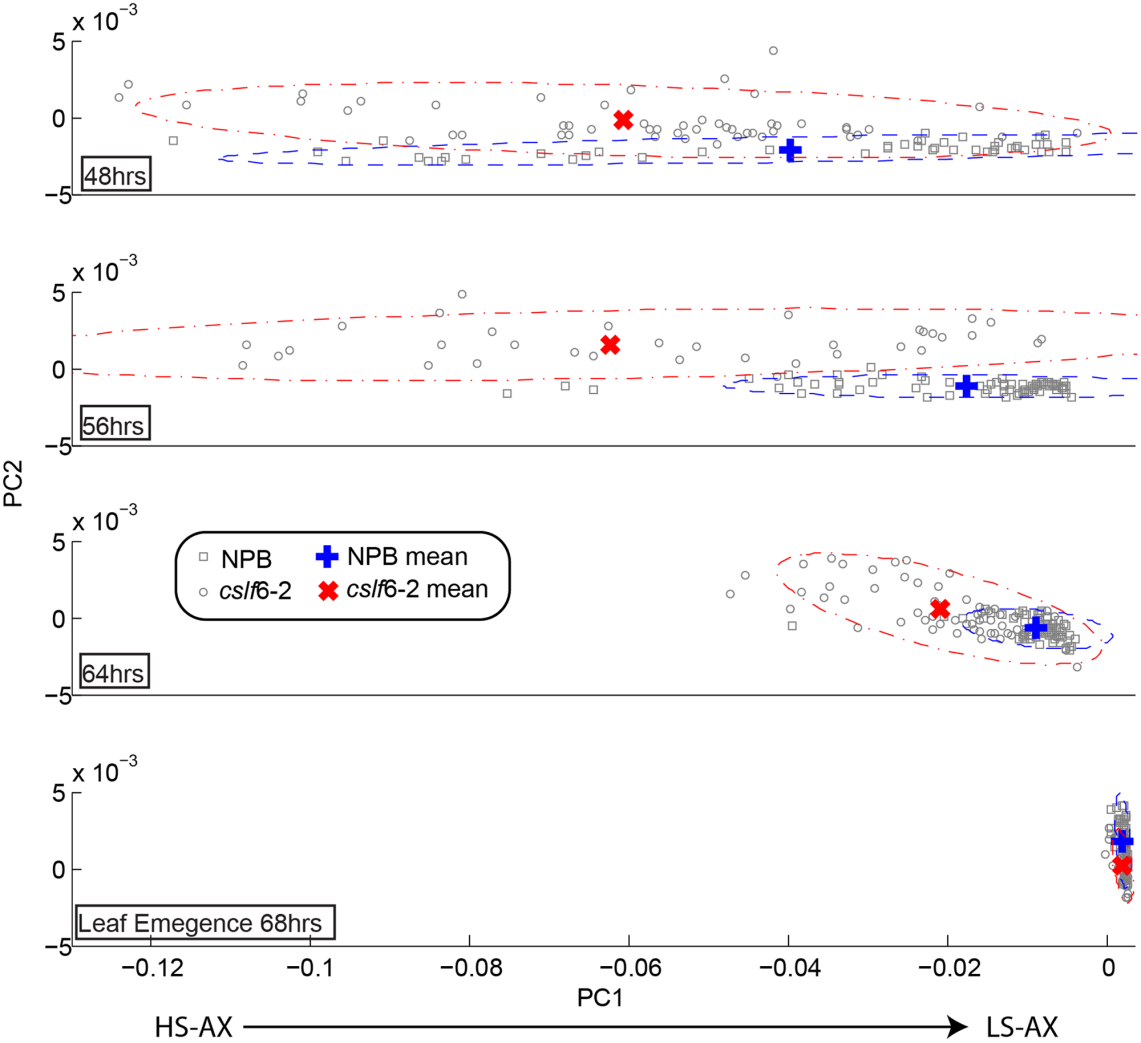
**Time course of arabinoxylan substitution pattern in coleoptile mesophyll cell walls.** Each panel represents the comparative map derived from principal component analysis (PCA) of second derivative absorption spectra of mesophyll cell walls in wild type (NPB) and *cslf6-2* mutant at different time points after seed germination. The x-axis (PC1) represents the range of substitutions going from highly substituted arabinoxylan (HS-AX; negative pc scores) toward low-substituted arabinoxylan (LS-AX; positive pc scores). Due to the biological variation, the mean of the pc scores was plotted to help visualize and interpret the results; confidence intervals at α = 0.05 for each population were drawn as ellipses.

Arabinoxylan is another major component in the primary cell wall of grasses and is also reported to play an important role in cell elongation. It is known that in growing tissues in grasses, arabinosyl substitutions in the arabinoxylan backbone are cleaved with the progression of cell elongation, going from a highly to a lowly substituted state (Carpita and Gibeaut, 1993). The application of FTM-IR spectromicroscopy to assay arabinoxylan’s degree of substitution has been previously validated (Robert et al., 2005; Saulnier et al., 2009). We used this technique in order to track the arabinoxylan substitution pattern in a time course developmental study of wild type and *cslf6-2* mutant seedlings. We specifically targeted mesophylls cells due to the fact these cells have been reported to contain the largest amount of MLG compared to other tissue types in coleoptiles (Carpita et al., 2001) and also the majority of MLG reduction occurred in the mesophyll cells (Vega-Sánchez et al., 2012). IR absorption spectra were taken from mesophyll cells in coleoptile sections at various time points. By performing PCA on pre-processed spectra for all time course stages, a loading plot was generated that has been previously correlated to the number of arabinoxylan substitutions (Supplementary Figure S2). We also measured Arabinose/Xylose content of whole wild-type coleoptiles with HPAEC at two time points to confirm decreased arabinoxylan branching as the coleoptiles matures (Supplemental Table S1). As shown in **Figure 3**, at the initial seedling growing stages, the *clsf*6-2 mutant appears to have a higher degree of arabinoxylan substitution compared to wild type. This trend continues until the last stage of leaf emergence (which signals the cessation of cell elongation in the coleoptile), where both wild type and mutant have similar arabinoxylan substitution profiles (**Figure 3**). The *cslf6-2* mutant has, on average, a much slower rate of progression from high to low arabinoxylan substitution than wild type during the initial stages of coleoptile development. These results suggest that lack of MLG affects the rate of arabinosyl side chain turnover in mesophyll cell walls. However, the overall substitution level of arabinoxylan in *cslf6* mutants is not altered at the end of the cell elongation phase.

### Cellulose Content and Organization

**FIGURE 4:**
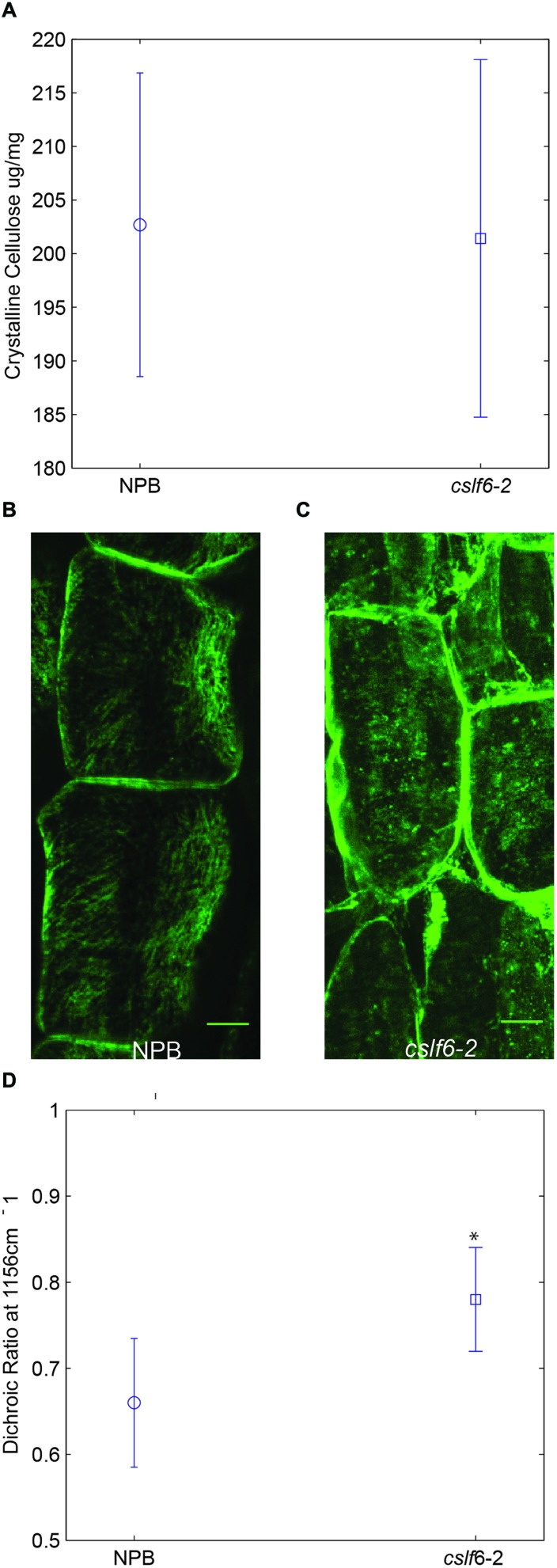
**Cellulose content and organization in wild type and *cslf6-2* knockout mutant. (A)** Crystalline cellulose content for 3-day-old wild type (NPB) and mutant (*cslf6-2*) seedlings. Z-stack of fluorescent confocal images depicting labeling of cellulose microfibrils with CBM3a in transverse sections of wild type **(B)** and mutant **(C)**. **(D)** Dichroic ratio of the glycoside bond in cellulose for wild type and mutant determined by polarized FT-MIR (*99.9% confidence level that the means of the dichroic ratios differ). *n* = 3–5 biological replicates. Scale bar = 10 mm.

Previous studies have suggested that MLG coats cellulose microfibrils in epidermal cell walls of maize coleoptiles (Carpita et al., 2001) and that it binds to cellulose irreversibly *in vitro* (Kiemle et al., 2014). Thus, we decided to test whether a lack of MLG has any effect on cellulose content or structure *in vivo*. We did not detect any significant differences in crystalline cellulose content between wild type and the *cslf*6-2 mutant (**Figure 4A**). We then addressed whether there are any changes associated with the structure or organization of cellulose microfibrils. Cellulose microfibrils are the main load-bearing components in plant cell walls and their orientation determines the direction of preferential wall extension (Carpita and Gibeaut, 1993; Suslov and Verbelen, 2006; Chan, 2012). To look at the orientation of cellulose microfibrils in coleoptile mesophyll cells along the axis of elongation, we first performed fluorescence microscopy studies using the cellulose-binding module CBM3a. This probe has been reported to bind specifically to crystalline cellulose (Blake et al., 2006). Sections of wild type seedlings were first treated with lichenase, a β-1,3-(1,4) endoglucanase, to remove MLG before immunolabeling to address possible masking issues. Fluorescent scans from multiple confocal planes were z-stacked and combined for maximum projection to generate an image of cellulose microfibrils. In wild-type coleoptiles, distinct cellulose microfibril bundles were easily visualized in mesophyll cells (**Figure 4B**). In contrast, fluorescent punctate structures were displayed in mutant cell walls, suggesting a lack of continuous structural arrangement of cellulose bundles (**Figure 4C**). It is known that cortical microtubules guide the deposition of cellulose microfibrils in plants (Paredez et al., 2006; Chan, 2012), however, using anti-tubulin immunofluorescence, we found no visual difference in the arrangement of microtubules for *cslf*6 compared to wild type (Supplementary Figure S3). In order to confirm the CBM3a labeling results, we performed a polarized FT-MIR analysis of longitudinal coleoptile sections. This method takes advantage of the birefringent nature of cellulose microfibrils and has been previously validated in oat coleoptiles and carrot cells (Morikawa and Senda, 1978; Mccann et al., 1993). By focusing polarized infrared light with a microscope objective through the cell wall, the net orientation of the cellulose microfibrils can be assayed by calculating the dichroic ratio in single mesophyll cells. Recording the absorption peak at 1156 cm^-1^ (representing the glycoside bond in cellulose) for both parallel and perpendicular polarizations allows for cellulose orientation determination in reference to the direction of cell elongation (Supplementary Figure S4). The dichroic ratio (parallel absorption/perpendicular absorption) of the cellulose glycoside bond for both wild type and mutant was less than 1, indicating an overall net preferential orientation perpendicular to cell elongation (**Figure 4D**). However, the statistically significant higher dichroic ratio in the *cslf6* mutant implies less preferential and more random cellulose microfibril orientation compared to wild type (**Figure 4C**). Taken together, these results show that lack of MLG directly affects normal cellulose deposition.

### Cell-Specific Changes Revealed by High Resolution FT-MIR Spectromicroscopy

Conventional FT-MIR microscopy can reveal compositional and structural information of a whole cell in a 50 μm by 50 μm area. However, coupling FT-MIR microscopy with a synchrotron light source allows even finer resolution, up to 3 μm, that can then be reconstituted into an image of multiple cells giving detailed spatial chemical information (Lasch et al., 2004; Holman et al., 2010; Lacayo et al., 2010). Since the *cslf*6 mutant does not appear to show dramatic differences in cell wall composition measured in bulk AIR samples with biochemical methods such as anion exchange liquid chromatography and glycome profiling, we investigated cell specific changes in primary walls of coleoptile mesophyll cells using synchrotron sourced FT-MIR.

**FIGURE 5:**
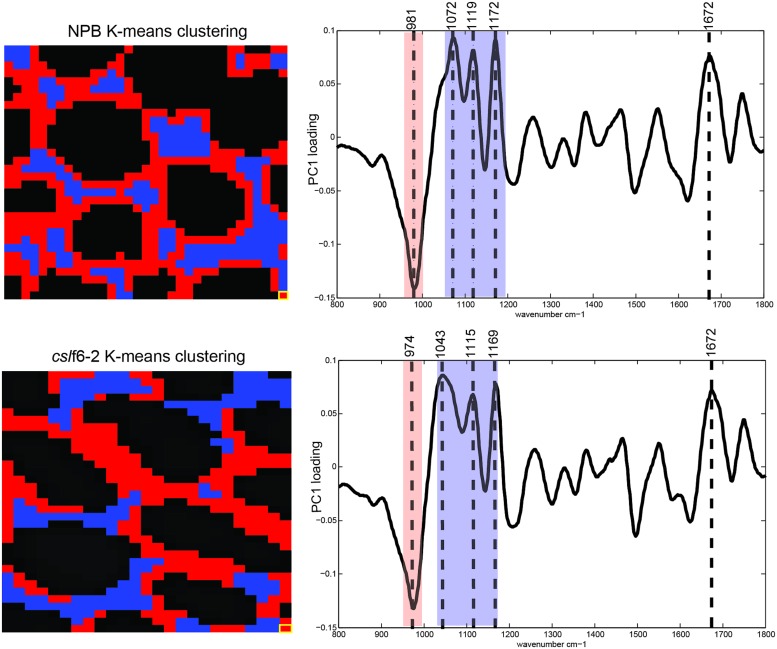
**Synchrotron FT-MIR analysis of wild type (NPB) and mutant (*cslf6-2*) cell walls.** Images are representative of three biological replicates for both NPB and *cslf6-2*. Each pixel (highlighted in yellow at bottom right corner) represents a 3 μm × 3 μm area. K-means clustering of spectra **(left)** and associated PC loadings **(right)** are shown. Blue and red pixels and wavenumbers represent cellulose and arabinoxylan spectral signatures, respectively. Note the more random organization of clusters associated with arabinoxylan and cellulose revealing less organization in the mutant, as well as the overall shift of wavenumbers to lower energy states in *cslf6-2* compared to wild type.

High resolution FT-MIR spectromicroscopy imaging was performed on 3-day-old coleoptile mesophyll cross sections. A schematic representation of the data analysis pipeline is shown in Supplementary Figure S5. Clustering analysis resulted in two distinct clusters for both wild type and mutant (**Figure 5**, Supplementary Figure S5). For wild type, we found two distinct areas: an area that surrounds the perimeter of the cell wall (red pixels) and the areas between cells (blue pixels). In the *cslf6-2* mutant, we observed an overall lack of spatial organization: there was no discrete cell wall perimeter as found for the wild-type cells (**Figure 5**). Looking at the spectroscopic difference between the two clustering groups, a very similar PC loading spectral profile was generated for both wild-type and mutant samples with four distinct cell wall signature peaks. In wild-type cells, the first peak at 981 cm^-1^ corresponds to arabinoxylan (Robert et al., 2005) while the other three peaks (1072, 1119, and 1172 cm^-1^) are cellulose specific signatures (Chen et al., 1997). In *cslf*6-2 cells, these four peaks were significantly shifted toward lower wavenumbers (974, 1043, 1115, and 1169 cm^-1^), while the reference amide band I peak at 1672 cm^-1^ (Lasch et al., 2004) remains the same for both samples. Lower wavenumbers correspond to lower vibrational/absorption energies. The shift of cellulose and arabinoxylan to lower energies in the mutant implies that, because these specific bonds do not require as much energy to vibrate/absorb compared to wild type, they are not as constrained by neighboring bonds or associations compared to the wild type. These results suggest that the main components of the MLG deficient mesophyll primary cell wall (cellulose and arabinoxylan) do not have as many bond associations or cross-links compared to the wild-type cell wall, thus possibly leading to a weaker cell wall.

### Mechanical Strength Testing

To test the effect of reduced MLG content on the mechanical properties of the 3-day-old seedling cell walls, we measured the bending stiffness or rigidity, calculated from a three point bending test of the lower 5-mm region of the coleoptiles. The lowest erect portion of the coleoptile in which the cells are fully expanded are of approximately uniform diameter (Ryden et al., 2003). We tested the mechanical properties with fresh mutant and wild-type seedlings, and found no significant difference in the bending rigidity (Supplementary Figure S6). This result shows that the mutant cell walls with turgor pressure have the same mechanical strength as wild type.

## Discussion

In grasses, MLG is a major component of the primary cell wall. In this study, we performed in-depth cell wall compositional and structural studies using the *cslf6* mutant to elucidate the role of MLG in grass primary walls. Although we were not able to find any striking modifications in cell wall composition, structural changes were revealed using spectromicroscopy analysis at the tissue and cell specific levels within the coleoptile mesophyll. These results suggest that *cslf6* mutant seedlings deficient in MLG have defective cellulose microfibril deposition and a possible lower rate of arabinoxylan turnover during cell expansion. At the single cell level, the structural changes observed for the *cslf6* mutant reveal a less organized and weaker cellulose association with surrounding primary components such as arabinoxylan. Interestingly, these structural changes do not seem to affect the overall mechanical properties of the mesophyll cell wall in the MLG-deficient mutant as revealed by our cell wall rigidity data. Previous data from analysis of maize coleoptile epidermal cell walls suggested that the majority of MLG in the primary wall is tightly associated with cellulose microfibrils, along with lowly substituted arabinoxylan and glucomannan (Carpita et al., 2001). More recently, *in vitro* binding studies demonstrated that MLG binds to both cellulose and arabinoxylan (Kiemle et al., 2014). Our results are in agreement with both of these studies: the absence of both cellulose-MLG and arabinoxylan-MLG interactions would provide an environment where arabinoxylan-cellulose dynamics are altered, as revealed by our polarization and FT-MIR synchrotron experiments. The defects in cellulose microfribril orientation in response to MLG deficiency strongly suggest that MLG may have a role in maintaining proper cellulose deposition. This idea is supported by the observation that primary cell wall *CesA* and *CslF6* (the MLG synthase) genes are co-expressed in rice (Supplementary Figure S7) and barley (Burton and Fincher, 2009), providing a functional link between cellulose biosynthesis and the accumulation of MLG in grasses.

In our previous work, we showed that mature stems are weaker in *cslf6* plants compared to wild type (Vega-Sánchez et al., 2012). We show here that in turgid fresh cells, however, there were no significant changes in the cell wall mechanical properties, which highlight the fact that the structural alterations we observed do not severely impact anisotropic cell expansion. This is in contrast to previous reports for other mutants that affect cellulose orientation in the primary cell walls, e.g., the *Arabidopsis csi* mutant (Li et al., 2012). We had previously reported that cell length is reduced by only 30% in *cslf6* coleoptiles (Vega-Sánchez et al., 2012), but otherwise seedlings developed normally, which is in agreement with our arabinoxylan substitution time course experiment where both *cslf6* and wild type reached the end of coleoptile cell elongation at the same time (68 h post-germination). Interestingly, Kiemle et al. (2014) found that removing MLG from maize and wheat coleoptiles does not induce wall extension based on a biomechanical cell wall creep test, which suggests that MLG does not act as tether between cellulose microfibrils. Although the three point bending experiments we carried out measure different properties (i.e., cell wall extensibility vs. strength or stiffness), our mechanical strength results are in agreement with the finding by Kiemle et al. (2014). Thus, we conclude that there is no experimental support for a role of MLG in cellulose microfibril tethering, as our results here and previously (Vega-Sánchez et al., 2012) complement those of Kiemle et al. (2014) and Carpita et al. (2001).

Our results, which showed cell-specific structural rather than compositional changes due to MLG deficiency, highlight the ability of the cell wall to compensate for missing primary components by macro structural re-arrangements. Based on our data, we propose a model to illustrate the role of MLG in grass primary cell walls: (1) the altered cellulose microfibril orientation suggests less organized and possibly shorter microfibrils in mesophyll cells; (2) the cellulose and arabinoxylan network surrounding the mesophyll cells is less organized and leads to fewer cellulose-arabinoxylan associations. In this context, MLG may be involved in the organization of cellulose microfibrils, specifically, in aiding in the formation of longer microfibril structures. It is known that MLG forms a gel-like structure (Fincher, 2009; Kiemle et al., 2014) and as such could act as an adhesive polymer that helps “stitch” cellulose fibers together.

The results presented in this study suggest that MLG is important for the arrangement of cellulose microfibrils in primary cell walls of coleoptile mesophyll cells. Unlike previous studies that have looked at the role of MLG upon chemical and enzymatic removal from the cell wall in wild-type plants, we have analyzed the effects on cell wall composition and structure in a mutant lacking this polysaccharide. Our results reveal significant but developmentally mild structural changes in 3-day-old seedlings in response to MLG deficiency. The use of high-resolution spectromicroscopy techniques, such as synchrotron FT-MIR, allowed us to probe single cells for discrete changes in cell wall structure that are not possible to achieve with bulk biochemical analyses.

## Author Contributions

AS-M and MV-S designed the research; AS-M, MV-S, SF-N, JF, and YV performed the research; AS-M, MV-S, SF-N, JF, and ZH analyzed the data; ZH, H-YH, and WW contributed analytical tools; and AS-M, MV-S, JH, HS, and PR wrote the paper.

## Conflict of Interest Statement

The authors declare that the research was conducted in the absence of any commercial or financial relationships that could be construed as a potential conflict of interest.

## Footnotes

1 http://www.canon.dk

2 http://imagej.nih.gov/ij/

3 http://infrared.als.lbl.gov/

4 http://www.ricearray.org/index.shtml
