# Supplementary material for: Structural characterization of a mixed-linkage glucan deficient mutant reveals alteration in cellulose microfibril orientation in rice coleoptile mesophyll cell walls
Source: Front Plant Sci. 2015 Aug 18;6:628. doi: 10.3389/fpls.2015.00628 (PMC4539472; doi:10.3389/fpls.2015.00628)
Supplement: Supplementary file 1 [file Data_Sheet_1.PDF]

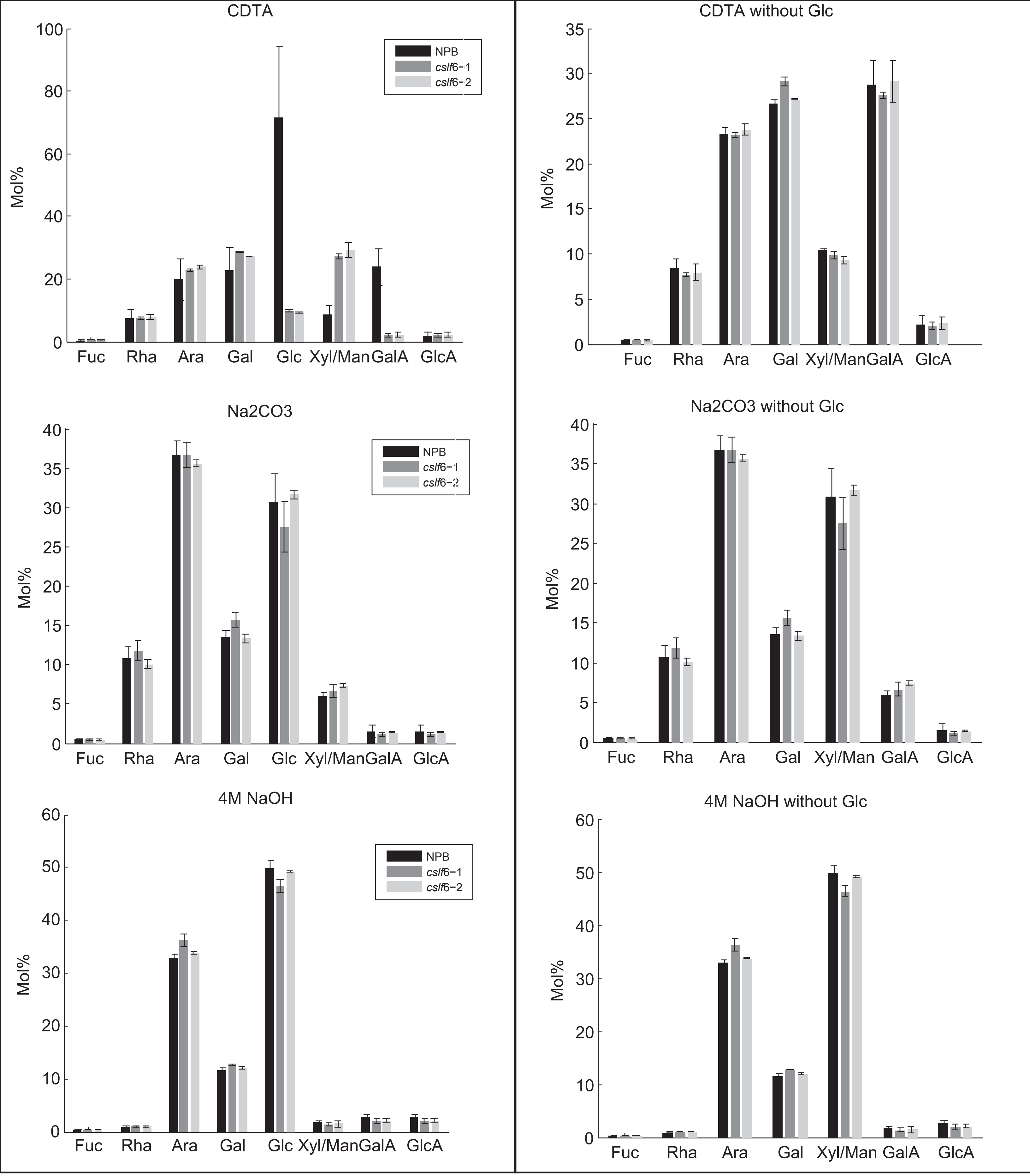

Supplementary Figure S1. HPAEC of sequential extraction of wildtype NPB and *cslf6* mutants. Cell wall composition recorded in mol% of all monosaccharides, implies compositional differences between wildtype and mutant. However, looking at mol% calculated with glucose removed, reveals no difference in the relative cell wall compostion between wildtype and mutant

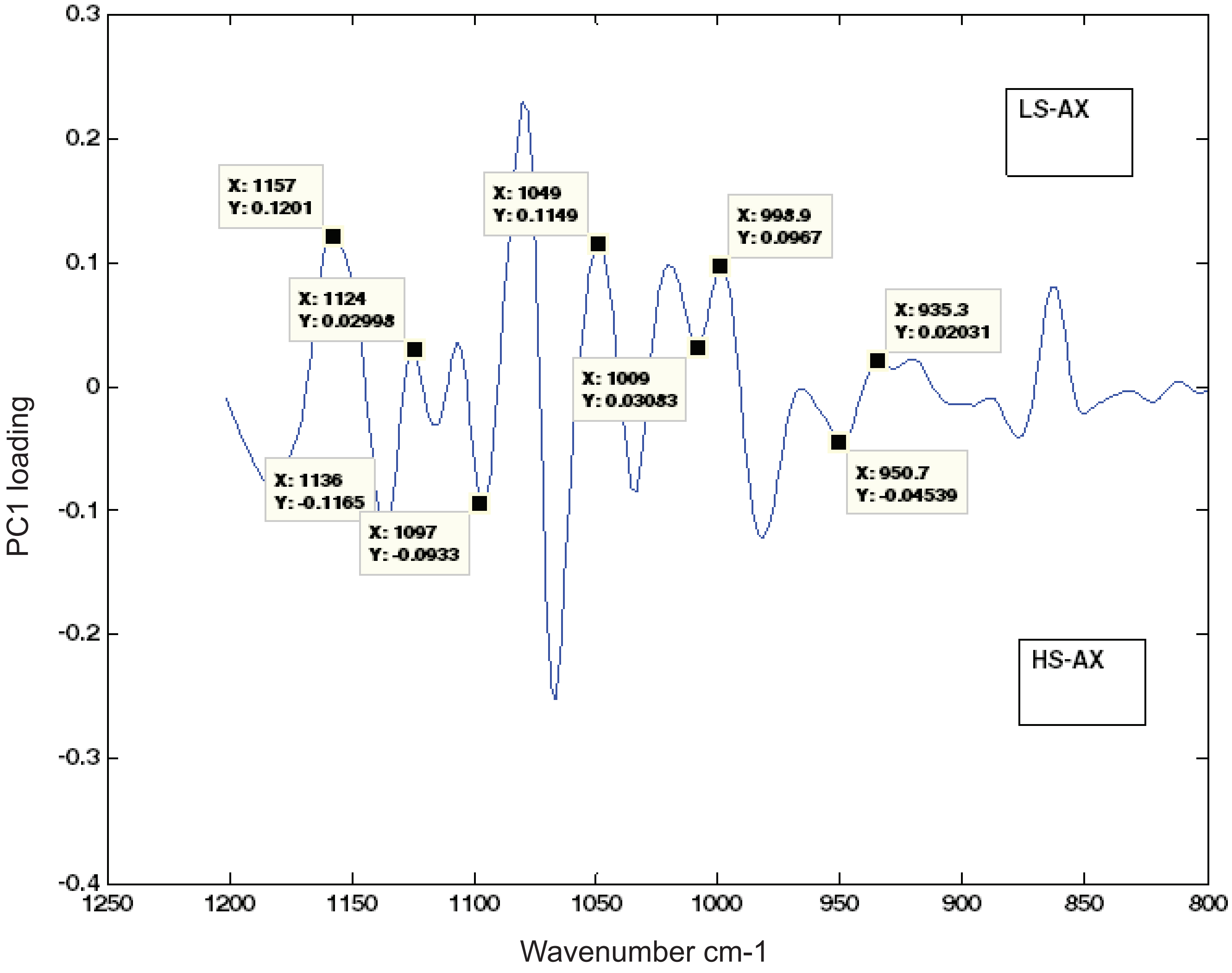

Supplementary Fig. S2. Principal component analysis of time-course development study of wild type NPB and *cslf*6-2 mutant tracking the arabinoxylan substitution pattern. This is the first principal component loading plot, with positive peaks characteristic of low substituted arabinoxylan (LS-AX), and negative peaks characteristic of highly substituted arabinoxylan (HS-AX).

Supplemental Table S1
4M KOH extracted samples: Arabinose/Xylose ratios

	Ara (mol%)	Xyl (mol%)	Ara/Xyl
60h seedlings	25.34 ± 0.66	29.80 ± 1.51	0.85
72h seedlings	23.47 ± 0.27	32.75 ± 1.46	0.72

Supplemental Table 1.Arabinose and Xylose content of wild type, NPB coleoptiles measured in mol% at 60h and 72h time points. This was measured using the 4M KOH fraction run on HPAEC. This confirms the reduction of arabinoxylan branching as the coleoptile matures. n=50.

(a)

(b)

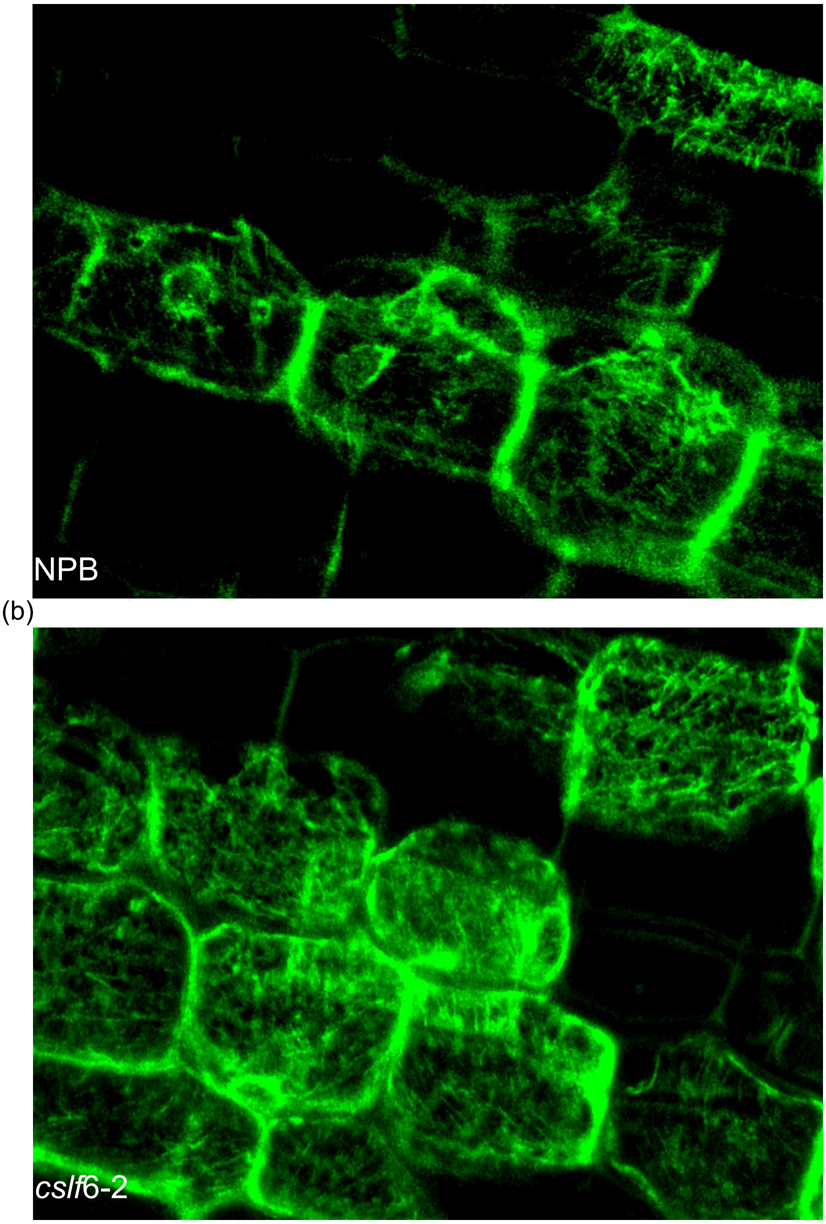

Supplementary Fig. S3. Immunolabeling of cortical microtubules with anti-α-tubulin antibodies. There appears to be no difference of labeling between lichenase treated wildtype(NPB) and *cslf*6-2 mutant mesophyll cells.

(a)

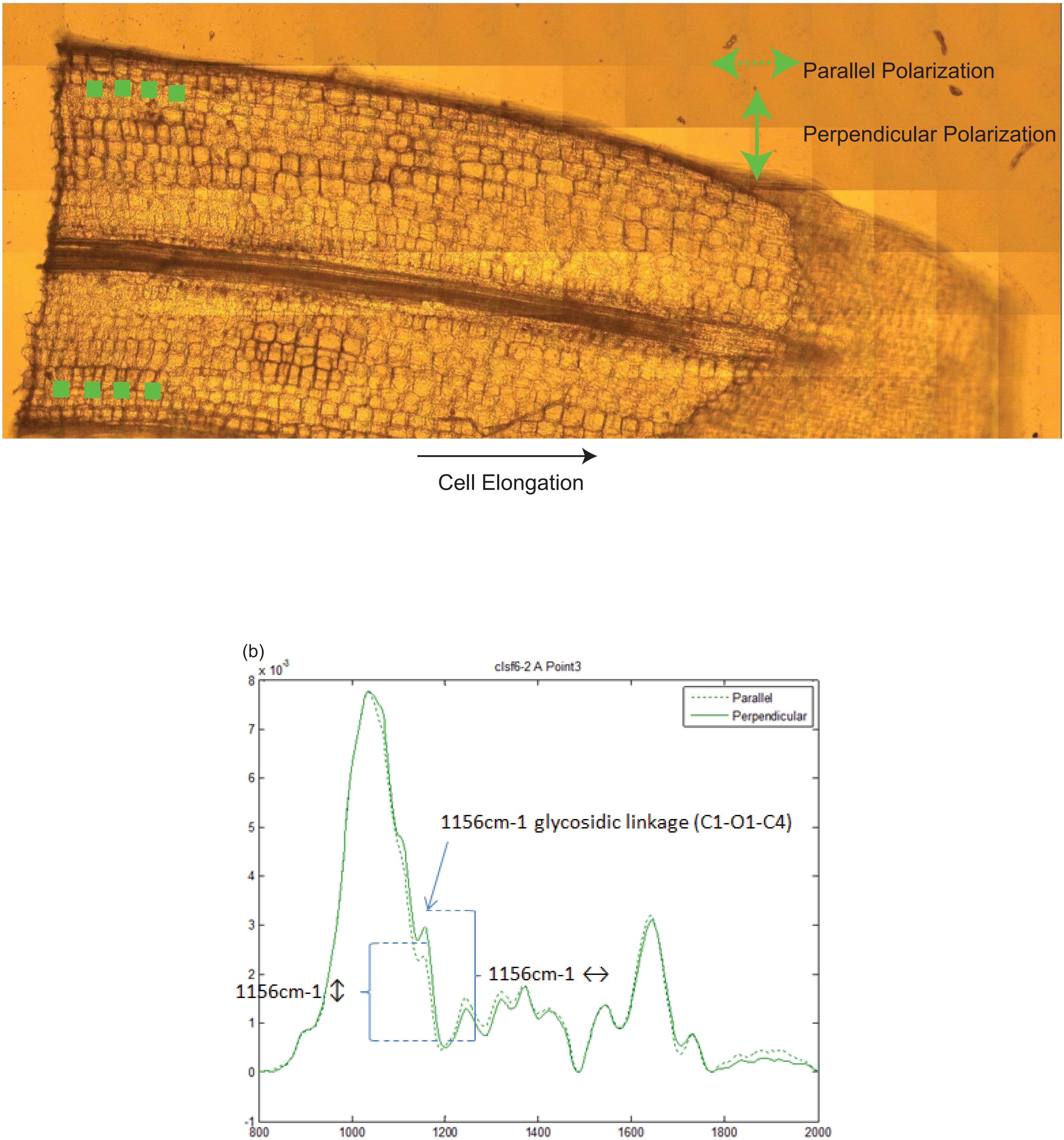

Supplementary Fig. S4. Example of an FT-IR polarization measurement.(a) Brightfield image of longitudinal section of 3 day old rice coleoptile with parallel and perpendicular directions to cell elongation indicated with 50 x 50um measurement areas marked in green. (b) Infrared absorption spectra of a mesophyll cell showing polarization sensitive peak 1156cm-1 representing the glycosidic bond in cellulose

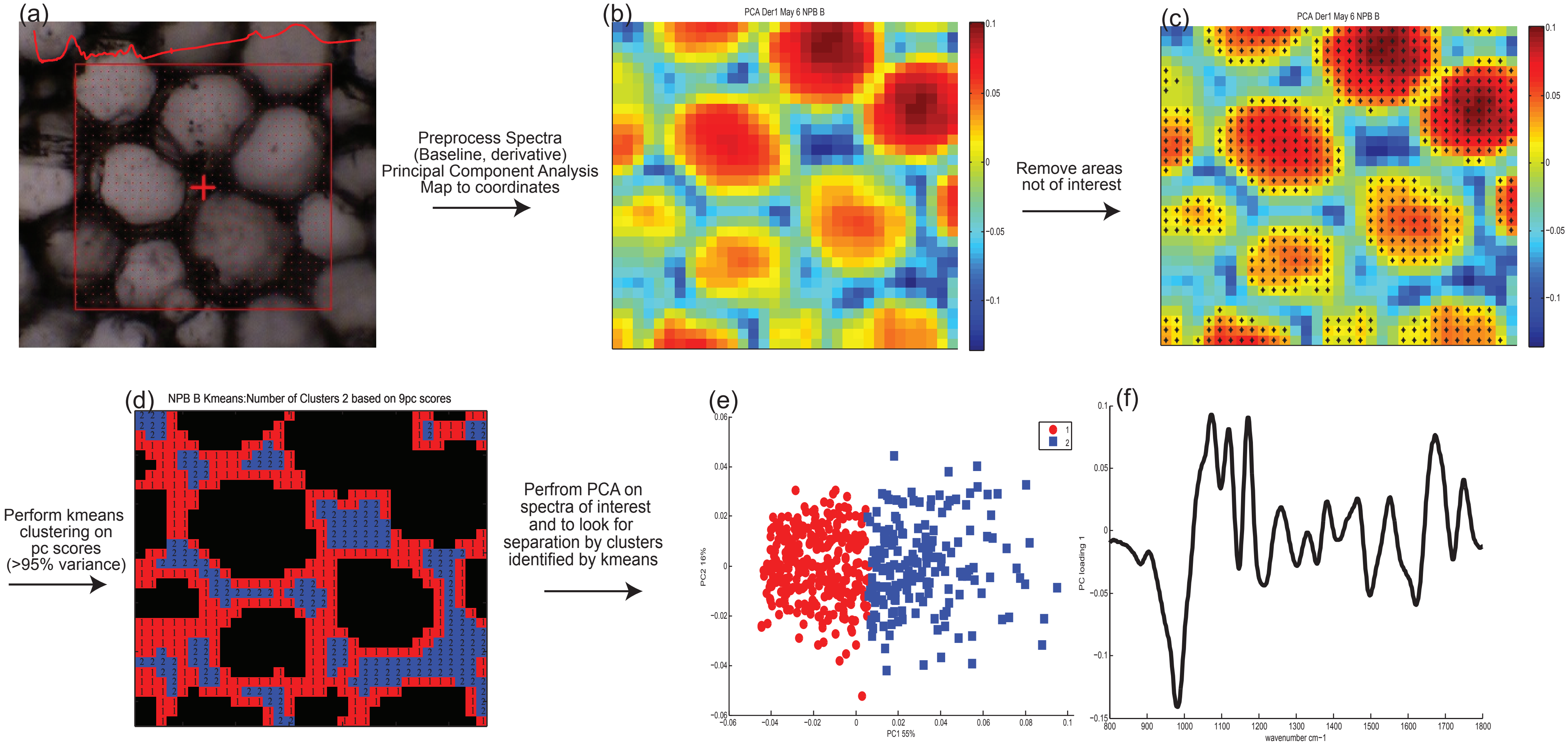

Supplementary Fig. S5: Schematic representation of high resolution spectromicroscopy data analysis stream. Spectra were taken at 3 um incremental steps from a sectioned sample (a). First pc scores were plotted according to spatial coordinates, generating an image resembling the mesophyll cells (b). Areas of interest were identified by first principal component scores: for example, PC1 scores greater than 0 were removed from the data set (c). The pc scores that represented 95% of the variation of the remaining areas of interest were then analyzed via a K-means clustering algorithm to search for biologically relevant grouping. Once the numbers of groups were defined and members identified (d), principal component analysis was performed on the first derivatives of the remaining areas. PC1 vs PC2 were plotted and grouped according to their cluster ID to identify which principal component separates the groups (e). Following this method, the first pc loading was used to describe the difference between groups (f) and therefore represents the chemical and structural differences reflected in the clustering according to spatial coordinates.

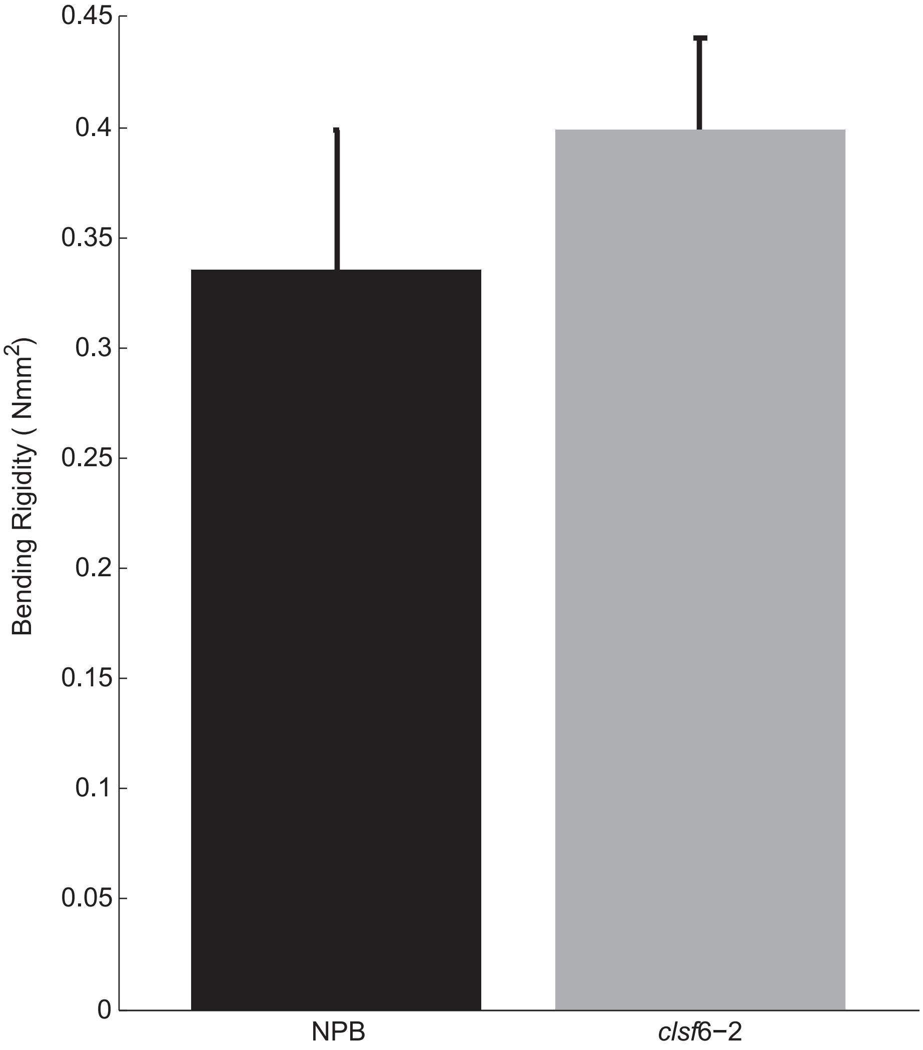

Figure. S6. Bending stiffness calculated using a three-point bending test in fresh wild type (NPB) and mutant (cslf6-2) three day-old seedlings. n= 10-20 samples

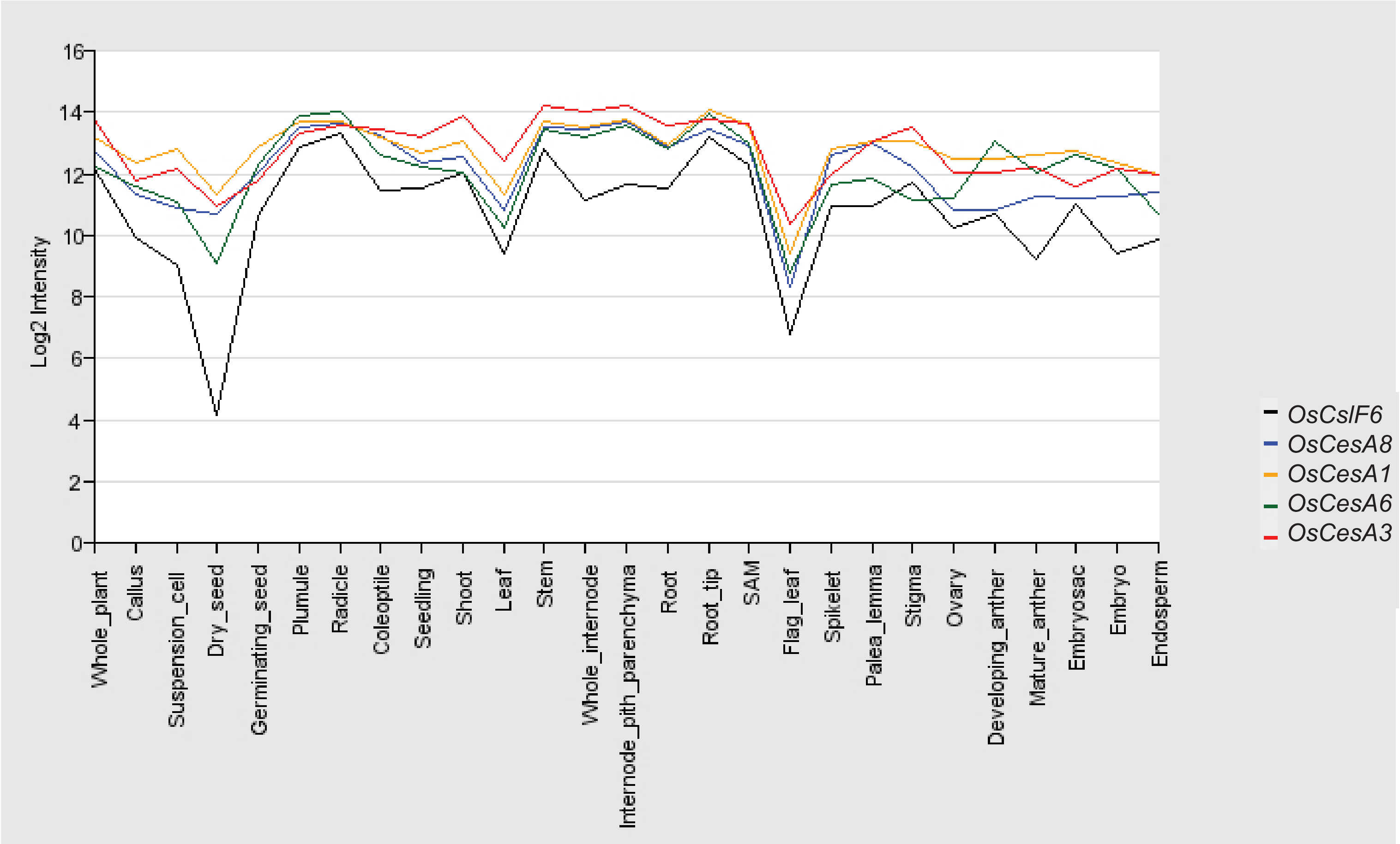

Supplementary Fig. S7. Coexpression analysis between *CslF6* and primary cell wall *CesA* (*Cellulose synthase*) genes in rice. The normalized expression values (Log2 values) for each of these genes throughout 27 rice anatomical stages is shown.
